# Status and Mechanism of Insecticide Resistance in German Cockroach (*Blatella germanica* L.) Worldwide: A Literature Review

**DOI:** 10.21315/tlsr2025.36.3.15

**Published:** 2025-10-31

**Authors:** Resti Rahayu, Intan Ahmad, Muhammad Zai Halifiah Sinaga, Risa Ukhti Muslima, Robby Jannatan

**Affiliations:** 1 Department of Biology, Faculty of Mathematics and Natural Sciences, Universitas Andalas, 25163 Padang, West Sumatra, Indonesia; 2 School of Life Sciences and Technology, Institut Teknologi Bandung, 40132 Bandung, West Java, Indonesia

**Keywords:** *Blattella germanica* L., Insecticide, Pest, Resistance, Worldwide

## Abstract

German cockroaches (*Blattella germanica* L.) are major residential pests, with reports of insecticide resistance emerging from numerous regions worldwide. This study aims to investigate the global distribution of insecticide resistance in German cockroaches, explore the underlying resistance mechanisms, identify the specific insecticides that have shown reduced efficacy and examine how resistance has developed globally. A literature review was conducted, collecting relevant publications from journal databases such as Google Scholar, Science Direct, Wiley Online Library and Oxford Academic Journal up to the year 2024. The keywords used in the search included “resistance,” “insecticide,” “Blattella germanica” and “German cockroach.” The review included studies that provided data from field strains using contact-based assays. In total, 102 studies on resistance spanning 23 countries across four continents were identified. Resistance has been reported against 60 different insecticidal active ingredients, primarily from the pyrethroid and organophosphate classes, with varying degrees of resistance noted. Very high levels of resistance (RR > 100) were mostly recorded for pyrethroids. The predominant resistance mechanism observed involved metabolic mechanisms, particularly the increased activity of cytochrome P450 enzymes, followed by esterases and glutathione S-transferases (GST). Target-site mechanisms were also reported, including knockdown resistance (kdr) (L993F) and resistance to dieldrin (Rdl) (A302S). The combined mechanisms of resistance result in broad-spectrum resistance and potential cross-resistance. This review highlights the critical need for ongoing surveillance of insecticide resistance in German cockroaches and emphasises the urgency of developing more effective pest management strategies to address the escalating challenge of resistance.

**HIGHLIGHTS**

- Insecticide resistance in the German cockroach (*Blattella germanica* L.) has emerged as a significant global concern, with confirmed reports from 23 countries across four continents.
- To date, resistance has been documented against at least 60 insecticidal active ingredients, with particularly high prevalence among pyrethroids and first-generation insecticides such as organochlorines and organophosphates.
- Resistance patterns exhibit substantial regional variability, even among adjacent localities, underscoring the critical need for ongoing, site-specific resistance monitoring.

## INTRODUCTION

Cockroaches are common household pests, spreading diseases and showing high adaptability (Bell *et al*. 2007). In addition to triggering allergic reactions in sensitive individuals and contaminating food, they pose serious public health risks and contribute to significant economic costs (Bonnefoy *et al*. 2008). Their behaviour of regurgitating during feeding can directly contaminate surfaces and facilitate pathogen transmission to humans (Solomon *et al*. 2016).

German cockroaches are the most widespread cockroach pests globally, predominantly inhabiting human residential buildings and seldom found in outdoor environments. Their social, medical and economic impacts are considerable (Lee *et al*. 2021), primarily due to their developed resistance to insecticides, which enables them to outcompete around 40 other pest cockroach species in residential settings (Tang *et al*. 2019). Population control currently relies heavily on the use of insecticides. However, this heavy reliance has led to a major issue: the emergence of insecticide resistance (Chai & Lee 2010; Rahayu *et al*. 2012; Fardisi *et al*. 2019). Resistance occurs through various mechanisms, including metabolic mechanisms (increased enzyme activity), target site mutations (alterations in insecticide binding sites), reduced insecticide penetration due to changes in the insect cuticle and behavioural alteration (Panini *et al*. 2016).

Based on these facts, this study aims to map the global distribution of insecticide resistance in German cockroaches by conducting a literature review. In addition to identifying the types of insecticides that have been reported to be resistant in German cockroaches, this study also evaluates the resistance mechanisms and reviews the development of German cockroach resistance. This study aims to provide a comprehensive overview of the development of resistance in German cockroaches and its implications for future pest control strategies.

## METHOD

Publications were collected through keyword searches using terms such as “resistance,” “insecticide,” “Blattella germanica” and “German cockroach” across various academic databases, including Google Scholar, ScienceDirect, Wiley Online Library and Oxford Academic Journal. The initial selection was based on a review of article titles and abstracts to assess relevance. Subsequently, all full-text articles were thoroughly reviewed to extract detailed data on resistance status, insecticide types, methods used, resistance ratios and resistance mechanisms. Only journal articles published up to 2024 were considered.

To provide a more accurate and relevant picture of the current resistance landscape, we focused exclusively on studies involving field strains that had not been subjected to prior insecticide selection or crossbreeding. Additionally, we included only studies that utilised contact-based bioassays, such as topical application and surface contact. This approach was chosen to minimise the variability caused by different testing procedures, enabling more comparable resistance ratios across regions and over time. This perspective is expected to offer a clearer understanding of the actual levels of resistance that pest management programs are currently facing.

## RESULT AND DISCUSSION

**FIGURE 1:**
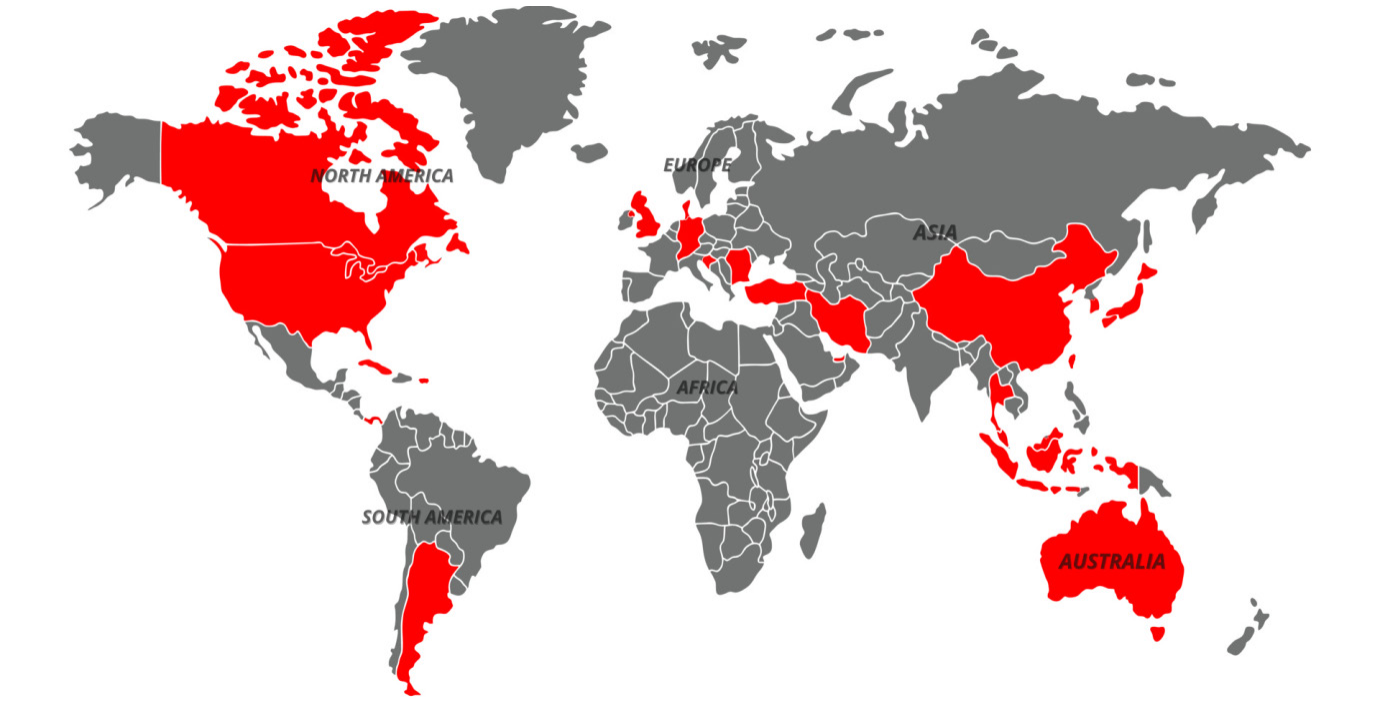
Global distribution of insecticide resistance reports in *Blattella germanica* L. (1953–2024). Red indicates countries with reported resistance cases, while gray represents countries with no available reports.

This review identified 102 studies of insecticide resistance in German cockroaches across 23 countries on 4 continents. Asia had the largest number of countries reporting resistance, while reports from Europe and Australia were more limited, and no data were found from Africa (Fig. 1). Reports of resistance in German cockroaches originate from diverse regions characterised by both tropical and subtropical climates. Based on the studies we found, cockroach samples have been collected from various urban residential environments, including apartments, residential areas, dormitories, hospitals, train stations, restaurants, malls, supermarkets, coffee shops, pubs, bakeries, food courts and other public facilities. These findings demonstrate that German cockroaches are exceptionally well adapted to human habitats. As they cohabitate closely with humans (Martin *et al*. 2015; Hulme-Beaman *et al*. 2016), this species is seldom found in areas distant from human activity (Valles 1996). The close association between humans and cockroaches has directly contributed to the increased reliance on insecticides, thereby accelerating the development of resistance.

Table 1 summarises reports of insecticide resistance in field strains of German cockroaches worldwide. The highest number of resistance cases was reported in the United States, with 45 studies, followed by Iran (16 studies), Denmark (6 studies) and Malaysia (5 studies). The earliest documented case of resistance occurred in Corpus Christi, Texas, where resistance to organochlorine, including active ingredients (AIs) chlordane, lindane and DDT, was recorded (Heal *et al*. 1953). Resistance to organophosphates (AI: diazinon) was first reported by Grayson (1961) in a strain from Owensboro, Kentucky. The initial instances of resistance to carbamates (AI: propoxur) were found in populations from seven locations in Louisiana (Bennett & Spink 1968). Schal reported pyrethroid resistance (AI: cypermethrin) in 1988, while Scott and Wen identified resistance to phenylpyrazole (AI: fipronil) in 1997. Resistance to neonicotinoids (AI: acetamiprid) was reported by Lee *et al*. (1999) and Wei *et al*. (2001) documented resistance to spinosyns (AI: spinosad) in the Opelika, Alabama cockroach population in 2001. Additionally, resistance to hydramethylnon and abamectin was reported by Scott (1991).

These historical data demonstrate how resistance can develop rapidly. For instance, although it was only marketed in 1947 (Ginnebaugh 1989), resistance levels to chlordane have been very high since the beginning of documentation (RR > 100) in 1953. Reports from various parts of the world also confirm that German cockroach resistance is a global phenomenon that has persisted for decades. Multistrain studies show high RR variation even within a single country, emphasising the importance of local surveillance. Extreme resistance is particularly dominant to the pyrethroid class, which has been widely and intensively used in cockroach control (Lee *et al*. 2022b), illustrating the accumulation of resistant individuals due to continued selection pressure on the same insecticide.

Our findings showed that some populations of German cockroaches remain susceptible to certain insecticides. Table 2 presents data on the frequency of insecticide resistance, with at least one strain reported to be susceptible. The pyrethroid and organophosphate classes dominate, considering that these two classes have the most types of insecticides reported with resistance cases in this study. Although d-Allethrin appears to show the lowest frequency of resistance, the limited number of tested strains suggests that caution is needed before drawing definitive conclusions. Meanwhile, fipronil with resistance tests spread across 23 studies from various countries, indicates that this insecticide is still more effective when compared to other insecticides, especially from the organochlorine class, which share the same mode of action (Table 4).

**FIGURE 2:**
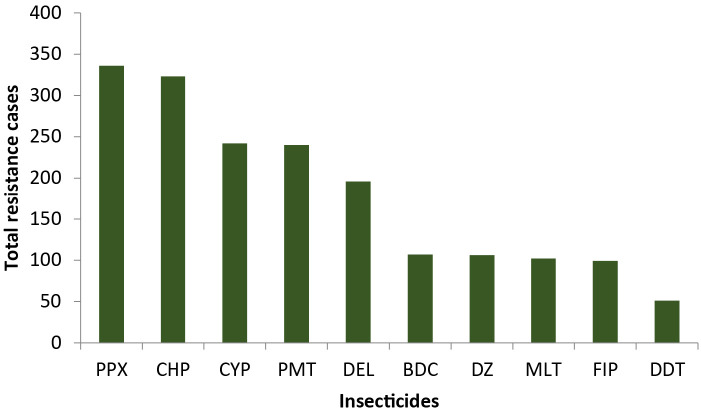
Top 10 insecticides ranked by the number of resistant *Blattella germanica* L. strains reported. Notes: PRX = Propoxur, CHP = Chlorpyrifos, CYP = Cypermethrin, PMT = Permethrin, DEL = Deltamethrin, BDC = Bendiocarb, DZ = Diazinon, MLT = Malathion, FIP = Fipronil, DDT = Dichloro-diphenyl-trichloroethane.

Fig. 2 illustrates the 10 insecticides with the highest number of resistant strains. First-generation insecticides, such as carbamates (propoxur, *n* = 336; bendiocarb, *n* = 107), organophosphates (chlorpyrifos, *n* = 323; diazinon, *n* = 106; malathion, *n* = 102) and DDT (*n* = 51), dominate the resistance reports. Among pyrethroids, cypermethrin (*n* = 242), permethrin (*n* = 240), and deltamethrin (*n* = 196) show the highest resistance levels. Resistance to fipronil (phenylpyrazole) is also notable, with 99 reports.

These data indicate that first-generation insecticides and pyrethroids show the most widespread and severe resistance in German cockroach populations, likely due to their prolonged and intensive use over time. The significant number of resistance cases associated with fipronil suggests that resistance is also emerging against newer-generation insecticides. This underscores the need for ongoing monitoring and the rotation of active ingredients in pest control programs.

Very high to extreme resistance to insecticides has been documented in various regions, with the top 10 instances detailed in Table 3. Multiple strains have been confirmed to possess diverse resistance mechanisms. These include metabolic resistance, which is mediated by enzymes (Wu *et al*. 1998; Wei *et al*. 2001; Hu *et al*. 2020), penetration resistance (Wu *et al*. 1998; Wei *et al*. 2001), or in combination (Wu *et al*. 1998; Wei *et al*. 2001). Meanwhile, strains Zo960302 and Ga021001 from Copenhagen, Denmark, which display extreme resistance to dieldrin, possess the Rdl mutation (A302S) at high frequencies, 0.97 and 1.0, respectively (Hansen *et al*. 2005; Kristensen *et al*. 2005).

Although several strains have no confirmed specific mechanism, those with very high resistance often exhibit cross-resistance to multiple insecticides, either within the same group (Öz *et al*. 2021) or across different groups (Jang *et al*. 2017). The BS-BG strain from Busan, South Korea, exhibits very high RR (> 200) not only to pyrethroids such as bifenthrin, esfenvalerate, cypermethrin, deltamethrin and permethrin but also to organophosphates, such as chlorpyrifos and chlorpyrifos-methyl. This broad-spectrum resistance suggests the involvement of metabolic or penetration mechanisms that contribute to cross-resistance across diverse insecticide classes, even with different modes of action (Jang *et al*. 2017).

Examining the history of insecticide use shows that several German cockroach populations have developed very high to extreme resistance after long-term and intensive exposure to insecticides (Choo *et al*. 2000; Rahayu *et al*. 2012; Wu *et al*. 1998). In contrast, the discontinued use of dieldrin has not lowered resistance levels, which remain persistent (Hansen *et al*. 2005; Kristensen *et al*. 2005). Since the *Rdl* mutation is strongly linked to dieldrin resistance and is present in the dieldrin-resistant strain, the continued existence of this resistance suggests stable genetic adaptations. Therefore, it is crucial to consider the possibility of ongoing resistance due to improper insecticide use when developing effective management strategies for German cockroach populations and preventing further cross-resistance.

**FIGURE 3:**
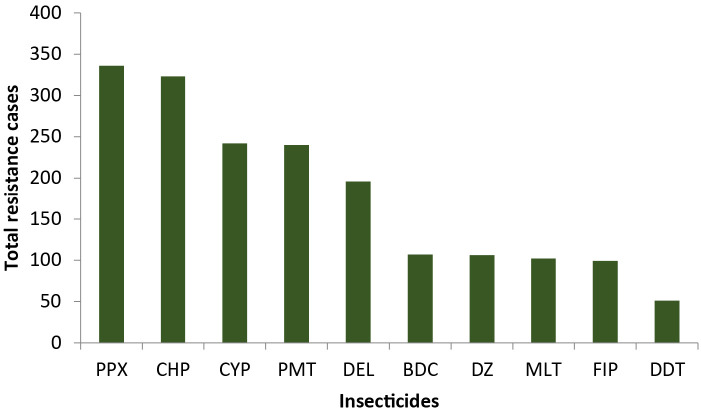
Distribution of enzymatic and target-site insecticide resistance mechanisms in *Blattella germanica* L. (*n* = 440).

Based on the total strains reported, the most common resistance mechanisms identified were metabolic resistance (82.5%), target-site resistance (16.9%) and penetration resistance (0.4%)—the latter of which is not included in Fig. 3. Metabolic resistance arises from increased biodegradation of insecticides due to heightened activity of detoxification enzymes (David *et al*. 2013). This review indicates that the highest levels of enzyme activity are associated with cytochrome P450 (*n* = 191), followed by esterase (*n* = 160) and glutathione S-transferase (GST) (*n* = 27).

All three enzymes function as detoxification agents, but they operate via different mechanisms. Esterase primarily contributes to insecticide resistance through the hydrolysis of ester bonds in insecticide molecules, especially pyrethroids and organophosphates. Additionally, esterase can sequester insecticides, binding and neutralising them without breaking them down. Cytochrome P450 (CYP) enzymes play a role in oxidative metabolism, converting lipophilic insecticides into more hydrophilic and less toxic metabolites through a process called monooxygenation. In contrast, GST detoxifies insecticides by conjugating them with glutathione, which facilitates excretion, and it is also involved in dehydrochlorination reactions (Nauen 2007).

In addition, genetic studies on resistant German cockroaches to several genes from the CYP family are positively correlated with resistance. Not only do they contribute to the metabolic pathway; CYP4G19, for instance, is reported to play a role in the production of cuticular hydrocarbons (CHCs), which are the primary components of the insect epicuticle and influence the penetration of insecticides into the insect’s body (Chen *et al*. 2020). Overexpression of CYP4G19 in the resistant strain was positively correlated with higher levels of CHCs, resulting in a penetration resistance mechanism in German cockroaches (Chen *et al*. 2020). The study by Tseng *et al*. (2024) also found that the CYP6K1 gene was overexpressed in resistant German cockroach strains, and silencing it reduced the level of resistance, leading to the conclusion of its role in pyrethroid resistance in German cockroaches. These findings highlight the multifaceted nature of insecticide resistance in German cockroaches, where defenses, both metabolic and structural, act synergistically to reduce the effectiveness of insecticides.

The target-site mutation L993F of the para-homologous sodium channel, known as knockdown resistance or kdr, was found in 47 test strains (Dong *et al*. 1998; Pridgeon *et al*. 2002; DeVries *et al*. 2019; Liu *et al*. 2022; Lee *et al*. 2022b; Tisgratog *et al*. 2023; Dashti *et al*. 2024), with 2 strains showing a novel mutation (L993S) that still needs further study (Liu *et al*. 2022). Meanwhile, the A302S mutation of the GABA-gated chloride channel known as dieldrin resistance (*Rdl*) was found in 26 strains (Hansen *et al*. 2005; Kristensen *et al*. 2005; Gondhalekar & Scharf 2012; Ang *et al*. 2013; Lee *et al*. 2022b; González-Morales *et al*. 2022; Tisgratog *et al*. 2023). As a note, *kdr* mutations are associated with pyrethroid and DDT class insecticides, while *Rdl* is associated with resistance to organochlorine class as well as phenylpyrazole. The findings of *kdr* and *Rdl* mutations across multiple countries highlight the global emergence of resistance to insecticide classes associated with target-site mutation. Addressing these resistance patterns is critical for maintaining the efficacy of vector control programmes, particularly since target-site resistance mechanisms are highly conserved and may be further selected under continued insecticide pressure.

Among the three resistance mechanisms identified, several studies have observed combinations of these mechanisms, including metabolic alongside target-site resistance (Hemingway *et al*. 1993b; Gondhalekar & Scharf 2012; DeVries *et al*. 2019; González-Morales *et al*. 2022; Lee *et al*. 2022b; Tisgratog *et al*. 2023), metabolic alongside penetration resistance (Wu *et al*. 1998), and combination of the three, which were reported in the Apyr-R strain from Opelika, Alabama, USA, from two separate studies (Wei *et al*. 2001; Pridgeon *et al*. 2002). Strains with those combination mechanisms showed very high levels of resistance, for instance, in fenvalerate (RR 825) (Wu *et al*. 1998), deltamethrin (RR 480) (Wei *et al*. 2001), and cypermethrin (RR 347) (DeVries *et al*. 2019). These findings highlight the synergistic effects of resistance mechanisms, which can lead to significantly elevated levels of cockroach resistance, even at high insecticide doses. Furthermore, the potential for cross-resistance limits the availability of alternative insecticide options.

Table 4 outlines 60 different active ingredients of insecticides associated with resistance cases in German cockroaches, categorised by their mode of action. The majority of resistance reports are linked to pyrethroids, followed by organophosphates and organochlorines. Many of these insecticides belong to classes that have been widely used in pest control but are now deemed obsolete by the World Health Organization (WHO), including EPN, acephate, bendiocarb, carbaryl and malathion (WHO 2020). Additionally, several of these substances are restricted, and the Rotterdam Convention regulates their distribution due to the significant risks to human health and the environment. Examples include chlordane, DDT, dieldrin, endosulfan, lindane, parathion, phorate and trichlorfon.

The prevalence of resistance and also the restricted regulation of insecticides from the organochlorine, organophosphate and carbamate classes, along with the widespread resistance to pyrethroids, highlights the urgent need to prioritise the use of newer insecticides with alternative modes of action. This approach is essential for effectively managing resistance and ensuring sustainable pest control.

It is worth noting that this review has several limitations. First, we exclusively included studies reporting resistance data obtained through contact-based bioassays, such as topical application and surface exposure. Consequently, data on the development of insecticide resistance in gel bait formulations are lacking. Additionally, information on behavioral resistance, which can only be observed through bait consumption or feeding assays, was not included. Furthermore, there is an imbalance in the amount of resistance data collected across different decades, which limits this study’s ability to provide a comprehensive understanding of long-term resistance trends in German cockroaches. These gaps highlight important areas for future research, emphasising the need for more systematic monitoring to accurately assess resistance dynamics over time and resistance mechanisms in this pest.

This study demonstrates that resistance in German cockroaches is a global issue, occurring regardless of climatic differences among countries. The resistance levels are primarily influenced by variations in pest management practices across different regions. As a result, German cockroach populations may exhibit significantly different resistance profiles even when collected from geographically adjacent areas. Accordingly, ongoing monitoring is crucial for accurately assessing the resistance profile in each region. This information is vital for developing effective pest control strategies and preventing further resistance development.

Novel insecticides, such as isocycloseram, may serve as promising alternatives for controlling German cockroaches due to their different modes of action compared to conventional insecticides (Lee *et al*. 2024). Boric acid is also an effective option; it is relatively non-toxic and has a non-specific mode of action, which allows it to remain effective even in cockroach populations that are resistant to neurotoxic insecticides, such as pyrethroids (Gondhalekar *et al*. 2021). Fungal-based biopesticides have also shown promise in combating resistance to conventional insecticides. A study by Zhang *et al*. (2022) indicated that resistance to insecticides can increase cockroaches’ susceptibility to fungi by altering their gut flora and gene expression. Additionally, plant-based bioinsecticides also show potential in managing pest resistance (Reda *et al*. 2017; Rahayu *et al*. 2020).

## CONCLUSION

German cockroaches have demonstrated remarkable adaptability to their host environment, contributing to their widespread distribution worldwide. The increasing use of insecticides to control German cockroach populations has accelerated the development of resistance through multiple mechanisms. The combination of mechanisms results in synergistic effects that not only increase resistance but also the incidence of cross-resistance, limiting alternative insecticide options.

German cockroach populations can exhibit very different resistance profiles, even from geographically adjacent areas. This fact highlights the need for continuous monitoring to assess resistance profiles in each region. The prevalence of resistance to insecticides, including organochlorines, organophosphates, carbamates and pyrethroids, underscores the urgent need to prioritise the development and use of newer insecticides with distinct modes of action. Further research is needed to explore behavioral resistance and other mechanisms in the German cockroach.

## Figures and Tables

**TABLE 1:** Summary of global reports on insecticide resistance in Blattella germanica L. (1953 to 2024)

No.	Country	Year^a^	Total strain^b^	Insecticide^c^	Assay^d^	Resistance ratio	Resistance category^e^	Resistance mechanism	References
	USA	1953	1	CHD, LND, DDT	TA	CHD (>100), LND (10–12), DDT (5–6)	Low–very high	**–**	Heal *et al*. (1953)
		1961	2	DZ, MLT	SC	DZ (2.5–5.8), MLT (NA)	Susceptible–medium	**–**	Grayson (1961)
		1965	1	CHD, DDT, DLD, LND, MLT, NL, CLC, B37344, B39007	TA	CHD (322.0), DDT (152.3), DLD (193.9), LND (23.5), MLT (5.57), NL (3.56), CLC (7.87), B37344 (28.7), B39007 (1.15)	Susceptible–very high	**–**	Ishii & Sherman (1965)
		1968	7	CHD, MLT, DZ, FT, PRX	TA	CHD (117.0–452.4), MLT (6.7–109.9), DZ (5.9–12.8), FT (8.2–10.6), PRX (1.9–14.7)	Low–very high	**–**	Bennett & Spink (1968)
		1971	17	CHD, MLT, DZ, PRX	SC	NA	Suspected resistance	**–**	Johnson & Young (1971)
		1982	1	CHD, MLT, DZ, CHP, PRX, BDC, ACE, FTT	SC	CHD (8.2), MLT (6.5), DZ (3.7), CHP (2.2), PRX (13.3), BDC (94.3), ACE (1.4), FTT (1.0)	Susceptible–very high	–	Nelson & Wood (1982)
		1985	2	DZ, CHP, MLT, PRX, BDC	SC	DZ (2.7–2.8), CHP (2.7–2.8), MLT (2.4–3.2), PRX (4.0–4.5), BDC (≥ 40)	Low–high	–	Robinson & Zungoli (1985)
		1988	6	PRX, BDC, CHP, CYP, DZ	SC	PRX (>100), BDC (>100), CYP (4.51), CHP (1.34), DZ (1.84)	Low–very high	–	Schal (1988)
		1989	45	DZ, CHP, ACE, MLT, PRX, BDC, PYR, ALT, PMT, PNT, FVL, CYF	SC	DZ (1–10), CHP (0–5), ACE (0–2), MLT (1–>60), PRX (1–>60), BDC (1–>60), PYR (0–>80), ALT (1–>100), PMT (0–>100), PNT (0–>80), FVL (0–>60), CYF (0–6)	Susceptible–very high	–	Cochran (1989)
		1990	2	BDC, CHP, CYP, DEL, FFT, MLT, PRX, PYR	TA	BDC (88.9–>277.8), CHP (3.4–4.6), CYP (4.9–7.8), DEL (0.2–3.3), FFT (1.8–5.2), MLT (5.4–24.2), PRX (5.2–5.7), PYR (6.1–9.5)	Susceptible–very high	Metabolic	Scott *et al. (*1990)
		1990	1	CHP, CH–O, CH–M, MLT, PRT, PRX, BDC, PYR, CYP	TA	CHP (21.6), CH–O (20.0), CH–M (11.5), MLT (>63.8), PRT (49.1), PRX (6.3), BDC (7.5), PYR (5.6), CYP (3.9)	Low–very high	Metabolic	Siegfried *et al*. (1990)
		1991	6	ABA	TA	ABA (0.5–10.0)	Susceptible–moderate	–	Scott (1991)
		1991	1	CYF, CYH, CYP, FVL, ESF, FLV, PMT, RES, SUM, TRA	TA	CYF (87.5), CYH (40.6), CYP (103.6), FVL (97.7), ESF (29.4), FLV (337.2), PMT (45.1), RES (102.6), SUM (113.8), TRA (72.2)	High–very high	Metabolic	Atkinson *et al*. (1991)
		1992	1	CYP	TA	122.6	Very high	–	Zhai & Robinson (1991)
		1992	1	BDC, CYP, CHP	TA	BDC (6.7), CYP (66.6), CHP (5.3)	Moderate–very high	–	Moss *et al. (*1992)
		1993	9	CHP, PRX	SC	CHP (1.4–58), PRX (0.1–4.2)	Susceptible–very high	Metabolic	Hemingway *et al*. (1993a)
		1993	8	CYF, FVL, CYP, L–CY	SC	CYF (0.5–5.4), FVL (0.03–4.2), CYP (3.0–12.5), L–CY (0.4–15.6)	Susceptible–high	Target-site, metabolic	Hemingway *et al*. (1993b)
		1993	1	CHP, PRX, CYP	TA	CHP (5.99), PRX (2.43), CYP (5.05)	Low–moderate	Metabolic	Prabhakaran & Kamble (1993)
		1993	7	CHP	TA	3.23–17.33	Low–high	–	Rust *et al. (*1993)
		1993	1	CYP, CHP, BDC, FTT, PRX, PYR	TA	CYP (29.1), CHP (40.7), BDC (6.7), FTT (3.4), PRX (1.6), PYR (37.5)	Low–high	–	Chapman *et al*. (1993)
		1994	1	CHP, CYP	SC	CHP (6–9), CYP (21–23)	Moderate–high	–	Hostetler & Brenner (1994)
		1995	2	PYR, ALT, CYP, PNT	SC	PYR, ALT, PNT (>100), CYP (>60)	Low–very high	–	Ross & Cochran (1995)
		1996	1	CYP, PMT, PRX, BDC, CHP	TA	CYP (28), PMT (12), PRX (17), BDC (46), CHP (7)	Moderate–high	Metabolic	Valles & Yu (1996)
		1997	6	FIP	TA	1.0–7.7	Low–moderate	–	Scott & Wen (1997)
		1997	1	CYP, CHP, L–CY	TA, SC	TA: CYP (82.2), CHP (5.22) SC: CYP (7.3), CHP (1.2), L–CY (1.5)	Low–very high	Metabolic	Scharf *et al. (*1997)
		1998	13	CYP	TA	5–214	Moderate–very high	Target-site	Dong *et al. (*1998)
		1998	1	CHP, PRX, PMT, CYP	TA	CYP (17.26), PRX (15.75), PMT (13.53), CHP (5.62)	Moderate–high	Metabolic	Park & Kamble (1998)
		1998	1	FVL	TA	825	Very high	Metabolic, penetration	Wu *et al. (*1998)
		1998	12	CYP, L–CY, PMT, PRX, CHP	TA	CYP (3–159), PMT (2–88), L–CY (4–55), PRX (5–33), CHP (3–19)	Moderate–Very High	Metabolik	Valles (1998)
		1999	13	L–CY	TA	2.9–66.6	Low–very high	–	Valles (1999)
		2001	1	PMT, DEL, IMI, SPI, FIP	TA	PMT (97), DEL (480), IMI (10), SPI (1.3), FIP (2.3)	Moderate–very high	Metabolic, penetration	Wei *et al. (*2001)
		2002	2	PMT, DEL	TA	PMT (46–54), DEL (47–50)	High–very high	Target-site	Pridgeon *et al*. (2002)
		2004	2	ABA, FIP	TA	ABA (2.5–6.8), FIP (8.7–9.3)	Low–moderate	–	Wang *et al*. (2004)
		2011	1	IND, PMT, CYP, DDT, FIP, DLD, CHP, PRX, IMI, ABA, CLF	TA	IND (5.88), PMT (77.22), CYP (86.54), DDT (>100), FIP (37.86), DLD (>100), CHP (25.64), PRX (13.91), IMI (7.55), ABA (1.28), CLF (5.70)	Moderate–very high	–	Gondhalekar *et al*. (2011)
		2012	1	FIP	TA	36.42	High	Target-site, metabolic	Gondhalekar & Scharf (2012)
		2013	14	IND	SC	NA	Suspected resistance	–	Gondhalekar *et al*. (2013)
		2017	6	PMT, CHP, PRX, IMI, FIP	TA	PMT (5.5–51.5), CHP (5.2–9.3), PRX (0.8–1.5), IMI (1.2–3.4), FIP (2.0–8.7)	Low–very high	–	Wu & Appel (2017)
		2017	2	IND, ABA, BOR, B–CY, BIF, L–CY, FIP, DNF, IMI, ACM, CTN, TMX, CLF, dan HYD	SC	NA	Suspected resistance	–	Fardisi *et al*. (2019)
		2018	6	PMT, CHP, PRX, IMI, FIP	SC	PMT (0.6–305.1), CHP (1.0–2.0), PRX (0.8–3.5), IMI (0.6–6.1), FIP (1.2–1.9)	Susceptible–very high	–	Wu & Appel (2018)
		2019	10	CYP, FIP	TA	CYP (59–347), FIP (6–23)	Moderate–very high	Target-site, metabolic	DeVries *et al*. (2019)
		2022	1	IND	SC	NA	Suspected resistance	Metabolic	Scharf *et al*. (2022)
		2022	5	FIP, CTN, IND, ABA, HYD, DEL	TA	NA	Susceptible–high resistance	–	Lee *et al*. (2022a)
		2022	5	FIP	TA	22.4–37.2	High	Target-site, Metabolic	González–Morales *et al*. (2022)
		2022	5	DEL, FIP, DDT, DLD	TA	NA	Suspected resistance	Target-site, Metabolic	Lee *et al*. (2022b)
		2024	2	ISO	TA	1.6–3.0	Low	–	Lee *et al*. (2024)
		2024	4	DEL	TA	NA	Suspected resistance	Metabolic	Tseng *et al*. (2024)
	Panama	1993	2	CHP, PRX	SC	CHP (1–15.4), PRX (2.3–3.2)	Low–moderate	Metabolic	Hemingway *et al*. (1993a)
		1993	2	CYF, FVL, CYP, L–CY	SC	CYF (1.1–5.9), FVL (1.7–3.5), CYP (3–24.5), L–CY (1.3–2.1)	Low–high	Metabolic	Hemingway *et al*. (1993b)
	Puerto Rico	2016	I	FIP, IND, HYD	TA	FIP (5.6), IND (23.21), HYD (3.9)	Low–very high	–	Ko *et al*. (2016)
	Canada	1977	7	CHD, PRX, CHP, DZ, MLT	TA	CHD (16.2–218.0), PRX (1.9–8.0), CHP (0.6–2.3), DZ (1.7–3.8), MLT (0.8–4.1)	Low–very high	–	Batth (1977)
	Cuba	2000	9	MLT, CHP, PI–M, PRX, CYP, L–CY, DEL	TA	MLT (0.17–25), CHP (0.5–11.8), PI–M (3.4–24.8), PRX (0.3–5.4), CYP (5.5–>306), DEL (12–250), L–CY (2.3–213)	Low–very high	–	Pantoja *et al*. (2000)
	Argentina	2017	2	DEL	SC	676.61	Very high	–	Mengoni & Alzogaray (2018)
		2022	1	B**–**CYP	SC	100	Very high	Metabolic	Boné *et al*. (2022)
	Japan	1988	1	ALT, TET, PMT, FVL, CYP, FPP, ETO, DDT, FTT, DZ, PRX, MET	TA	ALT (>23), TET (>46), PMT (46), FVL (31), CYP (36), FPP (19), ETO (40), DDT (>4.3), FTT (1.3), DZ (0.86), PRX (2.1), MET (1.5)	Susceptible–high	Metabolic	Umeda *et al*. (1988)
		1993	5	PMT, ETO, ALT, TET, RES, FVL, CYH, DEL, CYP, CPT, DDT, FTT, DZ, MLT, PRX	TA	PMT (61), ETO (20), ALT (34), TET (>30), RES (95), FVL (114), CYH (30), DEL (43), CYP (26), CPT (60), DDT (5.0), FTT (4.5), DZ (2.8), MLT (2.5), PRX (2.5)	Low–very high	Target-site	Mahmood (1993)
	UAE	1993	1	CHP, PRX	SC	CHP (1.4), PRX (1.6)	Low	Metabolic	Hemingway *et al*. (1993a)
		1993	1	CYF, FVL, CYP, L–CY	SC	CYF (1), FVL (3.7), CYP (5.1), L–CY (2.8)	Susceptible–moderate	–	Hemingway *et al*. (1993b)
	Malaysia	1996	12	PRX, BDC, CHP, CYP, PMT, DDT, PNT, DEL	TA	PRX (2.8–91.6), BDC (3.7–>60.0), CHP (2.0–7.6), CYP (1.2–22.5), PMT (1.0–14.6), DDT (>6.1–>6.5), PNT (13.3–51.9), DEL (5.9–23.6)	Low–very high	–	Lee *et al*. (1996)
		1998	5	PRX, CHP, CYP	SC	PRX (1.7–9.8), CHP (1.1–4.3), CYP (1.2–1.7)	Low–moderate	–	Lee (1998)
		1998	1	PRX, BDC, DEL	SC	PRX (1.3), BDC (3.1) DEL (2.1)	Low	Metabolic	Lee & Lee (1998)
		1999	23	PRX, BDC, CHP, FTT, PI–M, CYP, PMT, DEL, DZ, CH–M, MLT, CBR, ETP, BIF, ACM, DDT, END, DLD	SC	PRX (1.3–11.5), BDC (3.1–65.2), CHP (1.1–4.3), FTT (1.1–4.1), PI–M (1.3–3.1), CYP (1.2–3.6), PMT (1.3–14.5), DEL (1.1–2.9), DZ (1.0–3.7), CH–M (1.0–2.9), MLT (2.0–>275), CBR (2.5–9.8), ETO (1.3–3.2), BIF (1.0–2.2), ACM (1.0–2.1), DDT (1.3–40.7), END (1.1–2.5), DLD (1.2–4.4)	Susceptible–very high	Metabolic	Lee *et al*. (1999)
		2004	52	PRX, CHP, DEL, PMT	SC	PRX (1.0–>280), CHP (1.2–7.5), DEL (0.9–122), PMT (1.5–>280)	Susceptible–very high	Metabolic	Lee & Lee (2004)
	Indonesia	2009	4	PMT, CYP, D–AL	SC	PMT (0.91–95), CYP (1.63–3.63), D–AL (0.13–4.53)	Susceptible–very high	Metabolic	Ahmad *et al*. (2009)
		2012	6	PRX, PMT, FIP	TA	PRX (2.13–16.88), PMT (2.83–1013.17), FIP (2.11–44.72)	Low–extremely high	–	Rahayu *et al*. (2012)
		2019	2	PRX	TA	1.42–1.59	Low	–	Nurseha *et al*. (2019)
	Singapore	2000	10	DEL	TA	17.7–4,235	High–extremely high	–	Choo *et al*. (2000)
		2010	22	DEL, B–CY, PRX, CHP, FIP, IMI, IND	TA	DEL (4.5–468.0), B–CY (3.0–94.5), PRX (3.9–21.5), CHP (1.5–22.8), FIP (1.0–10.0), IMI (0.8–3.8), IND (1.4–5.3)	Susceptible–very high	Metabolic	Chai & Lee (2010)
		2013	6	DLD, FIP	TA	DLD (1.1–4.1), FIP (1.2–3.0)	Low	Target-site	Ang *et al*. (2013)
	Thailand	2023	7	FIP, DEL, IMI	TA	NA	Suspected resistance	Target-site, metabolic	Tisgratog *et al*. (2023)
	South Korea	2009	1	PPT, TET, CHP, FTT, PFF, CYP, PMT, DEL, L–CY	TA	PPT (0.7), TET (1.1), CHP (1.9), FTT (1.8), PFF (4.5), CYP (11.6), PMT (11.5), DEL (68.6), L–CY (111.1)	Low–very high	–	Chang *et al*. (2009)
		2010	7	BIF, CHP, CH–M, CYP, DEL, ESF, FT, PMT	TA	BIF (46.0–158.6), CHP (1.7–140.4), CH–M (2.0–7.5), CYP (15.9–88.1), DEL (60.9–160.0), ESF (19.5–270.2), FT (8.1–17.2), PMT (10.5–109.8)	Low–very high	–	Chang *et al*. (2010)
		2017	1	DEL, CH–M, PMT, ESF, BIF, CYP, CHP, FT	TA	FT (50), CHP (261), ESF (295), CYP (306), CH–M (312), DEL (450), PMT (569), BIF (624)	Moderate–very high	–	Jang *et al*. (2017)
	Taiwan	2005	60	CHP, PRX, CYP	TA	CHP (1.12–28.8), PRX (1.39–62.5), CYP (1.95–27.35)	Low–high	–	Pai *et al*. (2005)
		2020	24	DEL, PRX, FIP	SC	DEL (1–>817), PRX (0.66–7.13), FIP (1.47–3.76)	Susceptible–very high	Metabolic	Hu *et al*. (2020)
		2021	20	IMI, FIP, IND, HYD	TA	NA	Suspected resistance	Metabolic	Hu *et al*. (2021)
		2023	5	CYP, TET, PMT, DEL, CHP, FTT, PI–M, PRX, FIP, IMI	TA	NA	Suspected resistance (in permethrin)	–	Pai *et al*. (2023)
	China	1998	1	CYP	TA	14	High	Target-site	Dong *et al*. (1998)
		1999	1	PMT, DEL, CYP	TA	PMT (67.1), DEL (18.1), CYP (11.8)	High	Metabolic	Zhang *et al*. (1999)
		2015	4	DEL, CYP, ACE, PRX	SC	DEL (14.2–25.8), CYP (7.8–23.7), ACE (6.0–7.1), PRX (1.2–1.6)	Low–high	Metabolic	Liu *et al*. (2015)
	Iran	1997	5	B–CY, SUM, PMT, L–CY	SC	B–CY (1.3–1.5), SUM (3.1–7.8), PMT (2.2–3.0), L–CY (1.1–2.5)	Low–moderate	–	Ladonni (1997)
		2006	3	L–CY, PRX, PI–M	SC	L–CY (1.42–2.38), PRX (1.12–1.17), PI–M (0.75–0.77)	Susceptible–low	–	Kamyabi *et al*. (2006)
		2006	11	PMT, FIP	TA	PMT (8.6–17.7), FIP (0.96–2.6)	Low–high	–	Nasirian *et al*. (2006a)
		2006	11	FIP	SC	0.9–1.6	Susceptible–low	–	Nasirian *et al*. (2006b)
		2006	7	PMT, CYP, CYF	SC	PMT (5.3–23.7), CYP (2.9–20.3), CYF (2.4–11.4)	Low–high	–	Limoee *et al*. (2006)
		2007	2	PMT, DEL, CYP	SC	PMT (2.2–2.2), DEL (2.0–2.2), CYP (2.1–2.3)	Low	Metabolic	Enayati & Motevalli (2007)
		2007	7	PMT	SC	4.8–19.9	Low–high	Metabolic	Limoee *et al*. (2007)
		2009	11	PMT	SC	0.36–26.1	Susceptible–high	–	Nasirian *et al*. (2009)
		2011	3	PMT, CYP, BDC, CHP	TA	PMT (11.6–17.6), CYP (11.4–26.4), BDC (2.9–4.9), CHP (1.2–2.2)	Low–high	Metabolic	Limoee *et al*. (2011)
		2012	2	PMT, CYP,MLT, CHP	TA	PMT (3.2–3.4), CYP (3.2–6.2), MLT (5.2–6.2), CHP (2.2–2.4)	Low–moderate	–	Limoee *et al*. (2012)
		2016	5	BDC, CBR	SC	BDC (2.1–7.9), CBR (1.6–2.0)	Low–moderate	Metabolic	Salehi *et al*. (2016)
		2018	1	CYP	SC	3.4	Low	–	Shiravand *et al*. (2018)
		2020	2	MLT, PRX, L–CY		MLT (5.0–5.5), PRX (4.1–5.0), L–CY (1.6–1.8)	Low–moderate	–	Kakeh–Khani *et al*. (2020)
		2021	3	PMT	SC	3.3–6.2	Low–moderate	Metabolic	Ghaderi *et al*. (2021)
		2022	3	CYP, PRX, FTT	TA	CYP (7.6–10.9), PRX (6.2–10.5), FTT (11.4–16.7)	Moderate–high	–	Fazeli–Dinan *et al*. (2022)
		2024	8	CYP	SC	1–5.4	Susceptible–moderate	Target-site	Dashti *et al*. (2024)
	Turkey	2021	5	DEL, PMT, A–CY, L–CY	SC	A–CY (545–≥1000), DEL (16.7–≥1000), L–CY (9.0–≥1000), PMT (7.7–≥1000)	Moderate–extremely high	–	Öz *et al*. (2021)
	Australia	1968	3	DLD, LND, MLT	TA	DLD (17.6–41.6), LND (59.5–86.0), MLT (0.1–0.2)	Low–very high	–	Hooper & Goward (1968)
		1969	3	DDT	TA	1.0–9.6	Susceptible–moderate	–	Hooper (1969)
		1991	1	DEL	TA	20	High	–	Horwood *et al*. (1991)
	Bulgaria	1991	5	DDT, PRX	SC	DDT (1.85–3.76), PRX (1.4–11.9)	Low	–	Gecheva (1991)
	UK	1993	3	CYP, CHP, BDC, FTT, PRX, PYR	TA	CYP (11.6–2.4), CHP (1.1–4.0), BDC (2.3–7.9), FTT (1.3–3.7), PRX (2.3–10), PYR (53.5–103.0)	Low–very high	–	Chapman *et al*. (1993)
	Germany	1998	1	CYP	SC	18	High	Target-site	Dong *et al*. (1998)
	Denmark	1993	10	PMT, DEL, CHP, DZ	SC	PMT (1–57), DEL (2–31), CHP (1–4), DZ (1–2)	Low–very high	–	Jensen (1993)
		1993	3	CHP, PRX	SC	CHP (0.4–12.2), PRX (1.1–2.3)	Susceptible–high	Metabolic	Hemingway *et al*. (1993a)
		1993	3	CYF, FVL, CYP, L–CY	SC	CYF (1.2–10.4), FVL (0.5–4.6), CYP (2.5–18.5), L–CY (2.0–9.4)	Susceptible–high	Metabolic	Hemingway *et al*. (1993b)
		1998	4	CHP, PMT, DEL	TA	CHP (1.1–5.1), PMT (16.0–47.0), DEL (23.0–44.0)	Low–high	Metabolic	Spencer *et al*. (1998)
		2005	2	DLD	TA	15–1,270	High–extremely high	Target-site	Hansen *et al*. (2005)
		2005	7	DLD, FIP	TA	DLD (2–2,030), FIP (1–15)	Low–extremely high	Target-site	Kristensen *et al*. (2005)
	Croatia	2024	2	CYP, DEL, IMI, CLF	TA	NA	Suspected resistance	–	Šimunac *et al*. (2024)

**TABLE 2:** Resistance frequency of *Blattella germanica* L. to insecticides with at least one susceptible strain reported.

No	Insecticide^a^	Chemical class^b^	Resistance frequency (%)	Tested strains (*n*)
1	DDT	DDT	98.08	52
2	Fenitrothion	OP	97.37	38
3	Chlorpyrifos	OP	97.29	332
4	Chlorpyrifos-methyl	OP	96.88	32
5	Diazinon	OP	97.25	109
6	Pirimiphos-methyl	OP	91.43	35
7	Acephate	OP	82.00	50
8	Malathion	OP	92.73	110
9	Propoxur	CB	94.12	357
10	Deltamethrin	PY	97.51	201
11	Cyfluthrin	PY	86.57	67
12	Bifenthrin	PY	96.77	31
13	Fenvalerate	PY	76.12	67
14	Lambda-cyhalothrin	PY	96.92	65
15	Permethrin	PY	92.66	259
16	d-Allethrin	PY	75.00	4
17	Phenothrin	PY	86.44	59
18	Pyrethrin	NP	92.59	54
19	Fipronil	PPZ	78.57	126
20	Imidacloprid	NEO	80.56	36
21	Acetamiprid	NEO	95.65	23
22	Abamectin	AVM	88.89	9

Notes:

a Only insecticides with at least one susceptible strain reported are included in this table. Insecticides with 100% resistance across all tested strains were excluded.

b OC = Organochlorine; OP = Organophosphate; CB = Carbamate; PY = Pyrethroid; NP = Natural pyrethrin; PPZ = Phenylpyrazole; NEO = Neonicotinoid; AVM = Avermectin.

**TABLE 3:** Ten case reports of insecticide resistance in *Blattella germanica* L. with the highest resistance levels worldwide.

Country	Class	Insecticide	RR50	References
Singapore	Pyrethroids	Deltamethrin	4,235	Choo *et al*. (2000)
Denmark	Organochlorines	Dieldrin	2,030	Kristensen *et al*. (2005)
Denmark	Organochlorines	Dieldrin	1,270	Hansen *et al*. (2005)
Indonesia	Pyrethroids	Permethrin	1,013.17	Rahayu *et. al*. (2012)
Turkey	Pyrethroids	Deltamethrin, alpha-cypermethrin, lambda-cyhalothrin, permethrin	> 1,000	Öz *et al*. (2021)
USA	Pyrethroids	Fenvalerate	825	Wu *et al*. (1998)
Taiwan	Pyrethroids	Deltamethrin	> 817	Hu *et al*. (2020)
Argentina	Pyrethroids	Deltamethrin	> 676.61	Mengoni & Aalzogaray (2018)
South Korea	Pyrethroids	Bifenthrin	624	Jang *et al*. (2017)
USA	Pyrethroids	Deltamethrin	480	Wei *et al*. (2001)

**TABLE 4:** Classification of insecticide types associated with resistance events in *Blattella germanica* L. categorised by their mode of action (IRAC, 2016).

Main group	IRAC group	Class	Active ingredient*	Mode of action
Acetylcholinesterase (AChE) inhibitors	1A	Carbamates	Bendiocarb, Carbaryl, Propoxur	Inhibit AChE, causing hyperexcitation.
	1B	Organophosphates	Acephate, chlorpyrifos, diazinon, malathion, naled, profenofos, parathion, trichlorfon, azamethiphos, chlorpyrifos-methyl, fenitrothion, fenthion, pirimiphos-methyl, piridaphenthion	
GABA-gated chloride channel blockers	2A	Organochlorines	Chlordane, Endosulfan, Dieldrin, Lindane	Block the Gamma-aminobutyric acid (GABA)-activated chloride channel, causing hyperexcitation and convulsions.
	2B	Phenylpyrazoles	Fipronil	
Sodium channel modulators	3A	Pyrethroids	Alpha-cypermethrin, allethrin, beta–cyfluthrin, bifenthrin, cypenothrin, cyfluthrin, cyhalothrin, cypermethrin, beta–cypermethrin, d–allethrin, deltamethrin, etofenprox, esfenvalerate, flucythrin, fenpropathrin, fenfluthrin, fenvalerate, fluvalinate, lambda–cyhalothrin, permethrin, phenothrin, pyrethrins, resmethrin, sumithrin, tetramethrin, tralomethrin	Keep sodium channels open, causing hyperexcitation and, in some cases, nerve block.
	3B	DDT	DDT	
Nicotinic acetylcholine receptor (nAChR) competitive modulators	4A	Neonicotinoids	Acetamiprid, clothianidin, dinotefuran, imidacloprid, thiamethoxam	Bind to the acetylcholine site on nicotinic acetylcholine receptors (nAChRs), causing a range of symptoms from hyper-excitation to lethargy and paralysis.
Nicotinic acetylcholine receptor (nAChR) allosteric modulators	5	Spinosyns	Spinosad	Allosterically activate nAChRs, causing hyperexcitation of the nervous system.
Glutamate-gated chloride channel (GluCl) allosteric modulators	6	Avermectins	Abamectin	Activates glutamate-gated chloride channels (GluCls) allosterically, leading to paralysis.
Miscellaneous non-specific (multi-site) inhibitors	8D	Borates	Boric acid	Disrupting various physiological functions of insects, especially the digestive tract.
Uncouplers of oxidative phosphorylation via disruption of the proton gradient	13	Pyrroles	Chlorfenapyr	Interferes with oxidative phosphorylation in mitochondria by uncoupling the proton gradient required for ATP synthesis.
Mitochondrial complex III electron transport inhibitors – Qo site	20	Hydramethylnon	Hydramethylnon	Inhibits electron transport complex III, preventing the utilisation of energy by cells by binding to the Qo site.
Voltage-dependent sodium channel blockers	22A	Oxadiazines	Indoxacarb	Block voltage-dependent sodium channels, causing nervous system shutdown and paralysis.
GABA-gated chloride channel allosteric modulators	30	Isoxazolines	Isocycloseram	Nerve action (strong evidence that action at this protein complex is responsible for insecticidal effects).

Notes: The active ingredient of insecticide reported in cases of resistance in German cockroaches. Chlordecone, Bayer 37344, Bayer 39007, chlorpyrifos oxon, and metoxadiazone were excluded as they are not classified under any IRAC mode of action group.
